# E- and P-Selectins Are Essential for Repopulation of Chronic Myelogenous and Chronic Eosinophilic Leukemias in a Scid Mouse Xenograft Model

**DOI:** 10.1371/journal.pone.0070139

**Published:** 2013-07-26

**Authors:** Daniel Wicklein, Anna Schmidt, Vera Labitzky, Sebastian Ullrich, Peter Valent, Udo Schumacher

**Affiliations:** 1 Institute of Anatomy and Experimental Morphology, University Cancer Center, University Medical-Center Hamburg-Eppendorf, Hamburg, Germany; 2 Ludwig-Boltzmann Cluster Oncology, Vienna, Austria; 3 Department of Internal Medicine I, Division of Hematology and Hemostaseology, Medical University of Vienna, Vienna, Austria; Queen’s University Belfast, United Kingdom

## Abstract

In chronic myelogenous (CML) and chronic eosinophilic leukemia (CEL), neoplastic cells spread via the circulation into various extramedullary organs. As E- and P-selectin constitute the starting point for the leucocyte adhesion/invasion cascade, and CEL and CML cells share many properties with normal granulocytes, we investigated the role of these selectins in CEL and CML cell expansion and organ invasion in a xenotransplantation model using scid mice. Using two human leukemic cell lines (EOL-1 and K562), we were able to show that E- and P-selectins mediate leukemia cell tethering and adherence in a laminar flow assay. While E-selectin binding depended on sialylated carbohydrate moieties, P-selectin binding was completely (K562) or partially (EOL-1) independent of these carbohydrates indicating the involvement of non-canonical selectin ligands. In a xenograft model in scid mice, both cell lines invaded the bone marrow and other organs, formed chloromas, and ultimately produced an overt leukemia. In contrast, in E- and P-selectin knockout scid mice, the cells failed to show engraftment in 8 out of 10 animals and even if they did engraft, they produced only little organ invasion and chloroma formation. Together, these data suggest that E- and P-selectins play an important role in leukemic dissemination in CML and CEL.

## Introduction

Chronic myelogenous leukemia (CML) is a clonal hematopoietic neoplasm with an annual incidence of 1–2 per 100,000 comprising 5–10% of all cases of myeloid leukemias. In the chronic phase of CML, the malignant clone is largely dependent on BCR-ABL kinase activity [1], [2], [3], [4]. In line with this assumption, the disease can be successfully treated with BCR-ABL tyrosine kinase inhibitors (TKI) such as imatinib [5], [6]. However, many patients relapse after discontinuation of TKI therapy, even after having entered a complete molecular response [7], [8]. This phenomenon is best explained by resistance of leukemic stem cells (LSC) that are often quiescent cells and therefore cannot be all eliminated by TKI treatment [9], [10], [11]. These LSC reside in bone marrow stem cell niches where they occasionally enter the organ’s micro-circulation before being spread into the blood to enter new survival niches in the bone marrow and in various extramedullary organs [12], [13], [14].

Chronic Eosinophilic Leukemia (CEL) is a rare myeloproliferative neoplasm characterized by clonal expansion of eosinophils and typical organ damage [15], [16], [17], [18]. The neoplastic eosinophils in CEL often display PDGFRA fusion genes, resulting in constitutive tyrosine kinase activity [19], [20], [21]. Like CML, the disease can be successfully treated with imatinib [18], [22], [23].

Although CEL and CML LSC populate the entire bone marrow and also other vascularized organs over time, not much is known about the molecular mechanisms that are involved in the dissemination and homing processes underlying LSC dissemination through the blood stream in these leukemias.

Recent xenograft experiments indicated that E- and P-selectin play a major role in the metastatic cascade of colon and breast cancer [24], [25]. These studies suggest that cancer cells mimic the adhesion cascade of leukocytes to emigrate from the bloodstream in which they are unable to survive. As cancer cells use the same mechanisms as leukocytes do to leave the bloodstream, it seems obvious that leukemic cells which are malignant counterparts of normal leukocytes use the same mechanism to translocate from the circulation into tissues in various organs. Additionally, it was recently demonstrated that E-selectin is a crucial component of a hematopoietic stem cell (HSC) niche in the bone marrow, with E-selectin regulating HSC dormancy and self-renewal [26].

In the present study, we analyzed factors that may underlie the dissemination of CEL and CML cells during the dissemination step of disease evolution. As the leukocyte adhesion cascade is generally considered to start with the selectins [27] and E-selectin is part of a HSC niche which might be a potential LSC niche as well [26], we chose to use a recently established xenograft model of human CEL [28] to investigate the behavior of CEL and CML cells in E- and P-selectin deficient scid mice.

## Materials and Methods

### Cell Culture

The CEL cell line EOL-1, the CML cell line K562 (DSMZ, Braunschweig, Germany) and the control, pancreatic adenocarcinoma cell line PaCa 5061 (characterized in [29]) were cultured as previously described [28], [29].

### Animal Experiments

The methodology for carrying out the animal experiments was consistent with the UKCCR guidelines for the welfare of animals in experimental neoplasia [30]. The experiment was recommended and supervised by the institutional animal welfare officer and approved by the local licensing authority (Behörde für Soziales, Familie, Gesundheit, Verbraucherschutz; Amt für Gesundheit und Verbraucherschutz; Billstr. 80, D-20539 Hamburg, Germany) under the project no. G10/55.

All animals used were pathogen-free Balb/c severe combined immunodeficient (scid) or E-selectin −/− and P-selectin −/− scid mice (previously described [24]) aged 9–14 weeks with a weight of 25–30 g at the beginning of the experiments. The mice were housed in filter-top cages, provided food and water ad libitum and their condition was monitored daily. Apart from visible chloroma and paraplegia, the general condition of the animals was evaluated by a standardized in house scoring system based on movement/behavior, weight development, food and water intake and fur condition. The mice were killed by cervical dislocation after having been anesthetized by intraperitoneal injection of a weight-adapted dose (10 µl/g bodyweight) of a mixture of 1.2 ml Ketamin (Gräub AG, Bern, Switzerland), 0.8 ml Rompun (Bayer AG, Leverkusen, Germany) and 8 ml saline.

### Flow Cytometry

Unconjugated antibodies against sialyl Lewis a/CA19-9 (Abcam), Cutaneous Lymphocyte Antigen (HECA-452), sialyl Lewis x/CD15s (CSLEX1) BD Biosciences, Heidelberg, Germany) and corresponding mouse IgM or rat IgM isotype control, respectively (Dako, Glostrup, Denmark), were detected with allophycocyanin (APC) conjugated goat anti-mouse Ig (BD Biosciences).

Cells were analyzed using a FACSCalibur cytometer (BD Biosciences). For assessment of E- and P-selectin binding on human tumor cells, E- and P-selectin chimeric proteins (R&D, Wiesbaden, Germany) were complexed by incubation with goat anti-human IgG1-biotin (Sigma Aldrich, Hamburg, Germany) and subsequent addition of phycoerythrin (PE, Dako) or APC (BD) conjugated streptavidin before staining.

Neuraminidase treatment experiments were performed by incubating the cells in PBS with or without 10 mU Neuraminidase from *C. perfringens* (Roche Diagnostics, Mannheim, Germany) for 1 h at 37°C prior to staining.

### Laminar Flow Adhesion Assay

Laminar flow experiments were performed as previously described [31]. Applied shear rates were 0.25 dyn/cm^2^ and 0.5 dyn/cm^2^.

### Xenograft Mouse Model of Human CEL and CML using EOL-1 Cells and K562 Cells

The experiment was carried out with 10 scid and 10 E-selectin −/−, P-selectin −/− scid mice in each group. For injection, EOL-1 cells or K562 cells were washed and resuspended in saline at 2×10^7^ cells per ml. All mice received an intravenous injection of 2×10^6^ EOL-1 or K562 cells (in 100 µl saline), respectively. The animals were controlled for development of symptoms of leukemia and/or visible chloroma each day. Mice with severe symptoms (in most cases apathy or paraplegia) were euthanized immediately.

### Immunohistochemistry

Immunohistochemistry using sections of paraffin-embedded tissues was carried out as previously described [28], [32].

### DNA Extraction and Real-time PCR for Detection of Human Leukemic Cells

DNA extraction from murine blood and quantification of human leukemia cells by real-time polymerase chain reaction (PCR) were performed as previously described [33]. For the quantification of human leukemia cells from bone marrow, 60 ng of DNA isolated from the animals’ bone marrow were used as template.

### Statistical Analyses

Statistical Analyses (Log-rank tests for the Kaplan Meier survival curves and Mann Whitney tests for leukemia cell numbers in blood and bone marrow) were performed using GraphPad Prism 5 (GraphPad, La Jolla, CA, USA). Tests used are given for each P value in the results section. Results were considered statistically different when the P value was below 0.05.

## Results

### Selectins Bind to Human CEL and CML Cells

**Figure 1:**
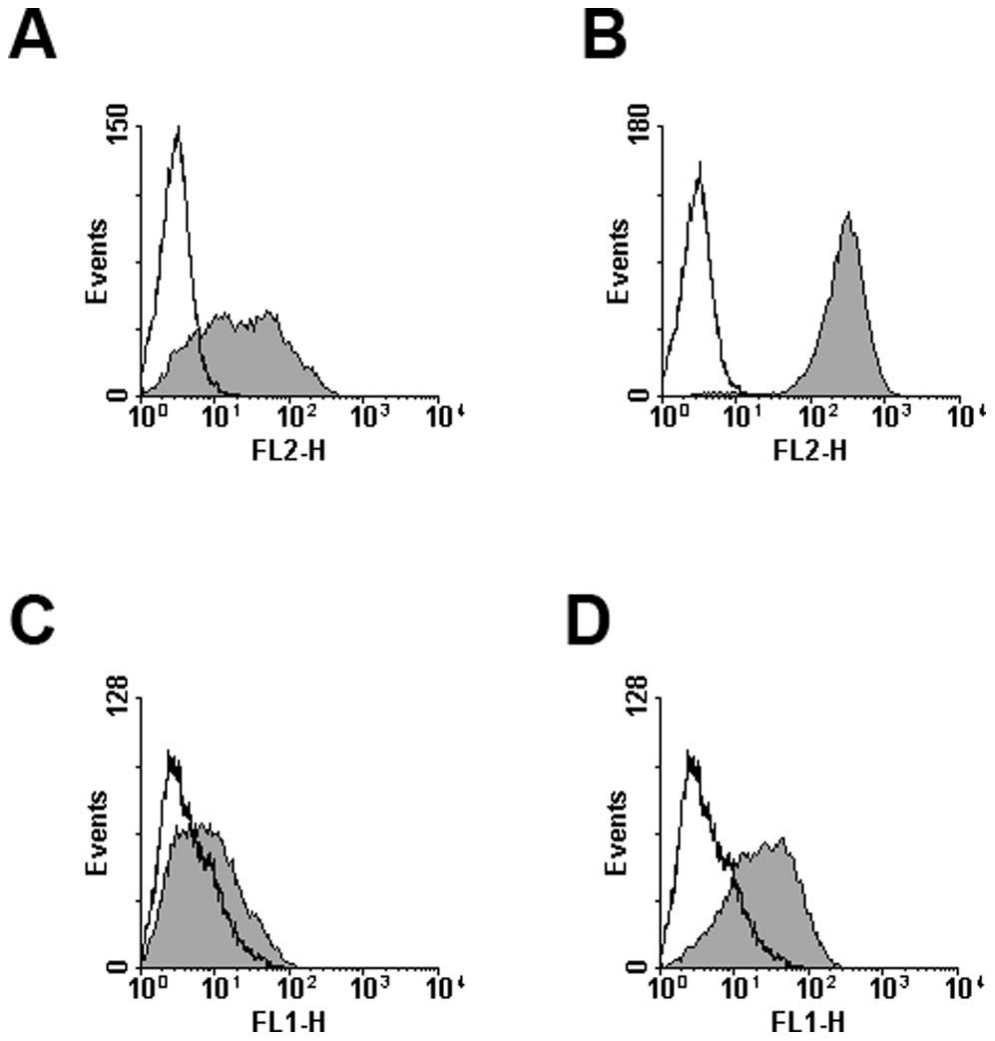
Human EOL-1 cells and K562 cells bind to E- and P-selectin *in vitro*. Binding of human E- and P-selectin to human CEL and CML cells was analyzed by flow cytometry. Given in the histograms are the fluorescence signal (FL-1 for AlexaFluor488 or FL-2 for phycoerythrin) and event number, selectin binding is represented by the filled curves, controls by the open curves. Cell lines and selectins used were: EOL-1 and human E-selectin (**A**), EOL-1 and human P-selectin (**B**), K562 and human E-selectin (**C**) and K562 and human P-selectin (**D**). All experiments were repeated twice, representative results are shown.

Binding of human E- and P-selectin to the human cell lines EOL-1 and K562 was investigated by flow cytometry. EOL-1 cells were found to bind to human E-selectin (**Figure 1A**) and human P-selectin (**Figure 1B**). K562 cells displayed weak human E-selectin binding (**Figure 1C**) and moderate human P-selectin binding (**Figure 1D**).

To investigate whether E- and P-selectin mediate tethering or adherence of human CEL and CML cells under shear stress (more closely reflecting *in vivo* conditions than the static incubation in the flow cytometry experiments), laminar flow adhesion experiments with both cells lines, EOL-1 and K562, were performed. Flow channels were coated with human and murine selectins, respectively.

**Figure 2:**
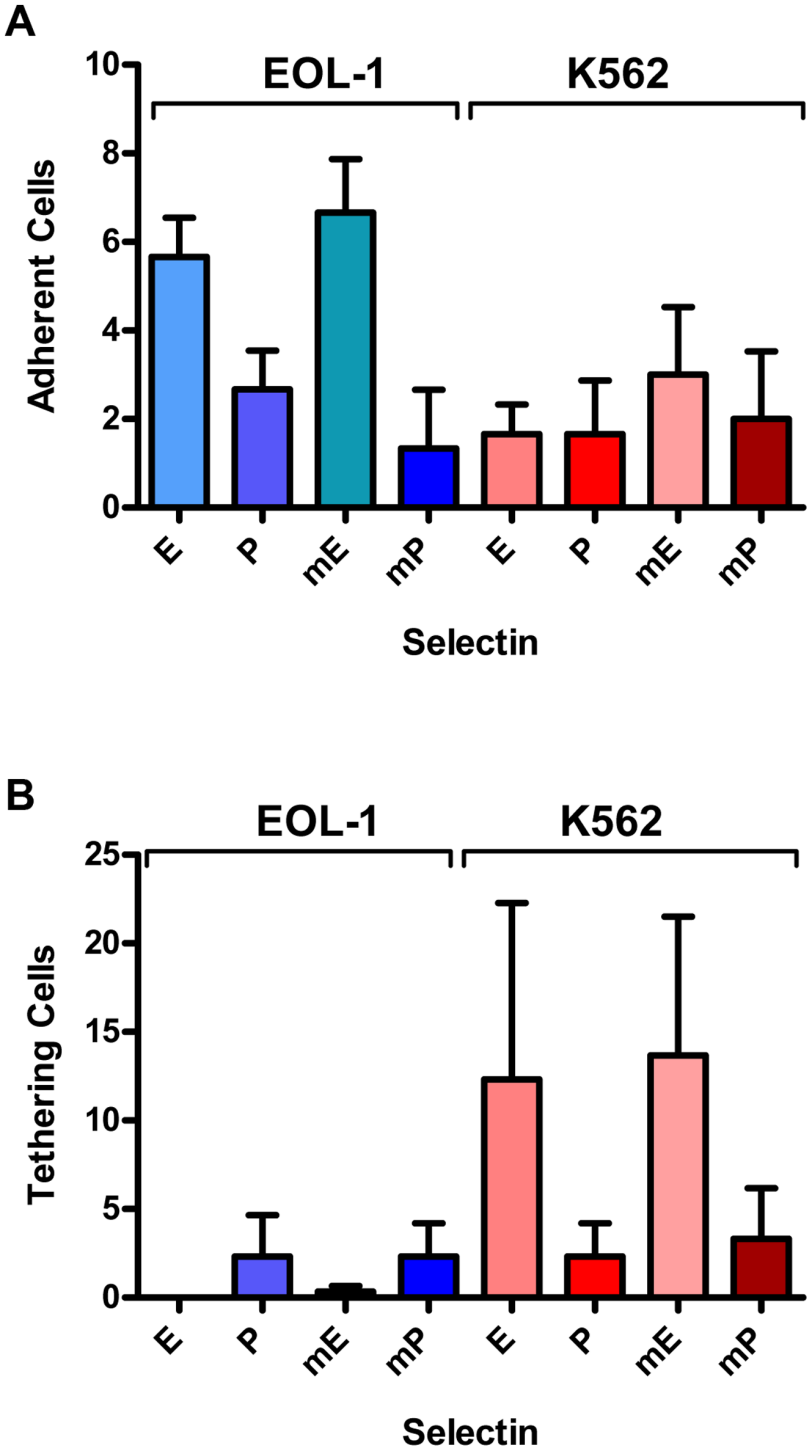
Human EOL-1 cells and K562 cells cells show tethering and adhesion to E- and P-selectin in laminar flow assays. Interactions of human CEL and CML cells from culture under laminar flow (0.25 dyn/cm^2^) in selectin coated channels. The cell lines EOL-1 and K562 were tested for adherence (**A**) or tethering (**B**). Given are the numbers and corresponding standard deviations of adhering and tethering cells, respectively. Channels were coated with human E-selectin (E), human P-selectin (P), murine E-selectin (mE) or murine P-selectin (mP). No adhering or tethering cells were observed in Fc control coated channels. All experiments were done in triplicates.

Both cell lines showed only weak adhesion to human and murine P-selectin. K562 cells displayed a similarly weak adhesion to both human and murine E-selectin (**Figure 2A**), while EOL-1 cells showed a stronger adhesion to human and murine E-selectin than the K562 cells.

Both cell lines showed only weak tethering in (human and murine) P-selectin coated channels. Almost no tethering EOL-1 cells were observed in channels coated with human or murine E-selectin, whereas K562 cells displayed strong tethering on human and murine E-selectin (**Figure 2B**).

### Selectins are Essential in Leukemia Cell Engraftment in a Xenograft Model of Human CEL with EOL-1 Cells

**Figure 3:**
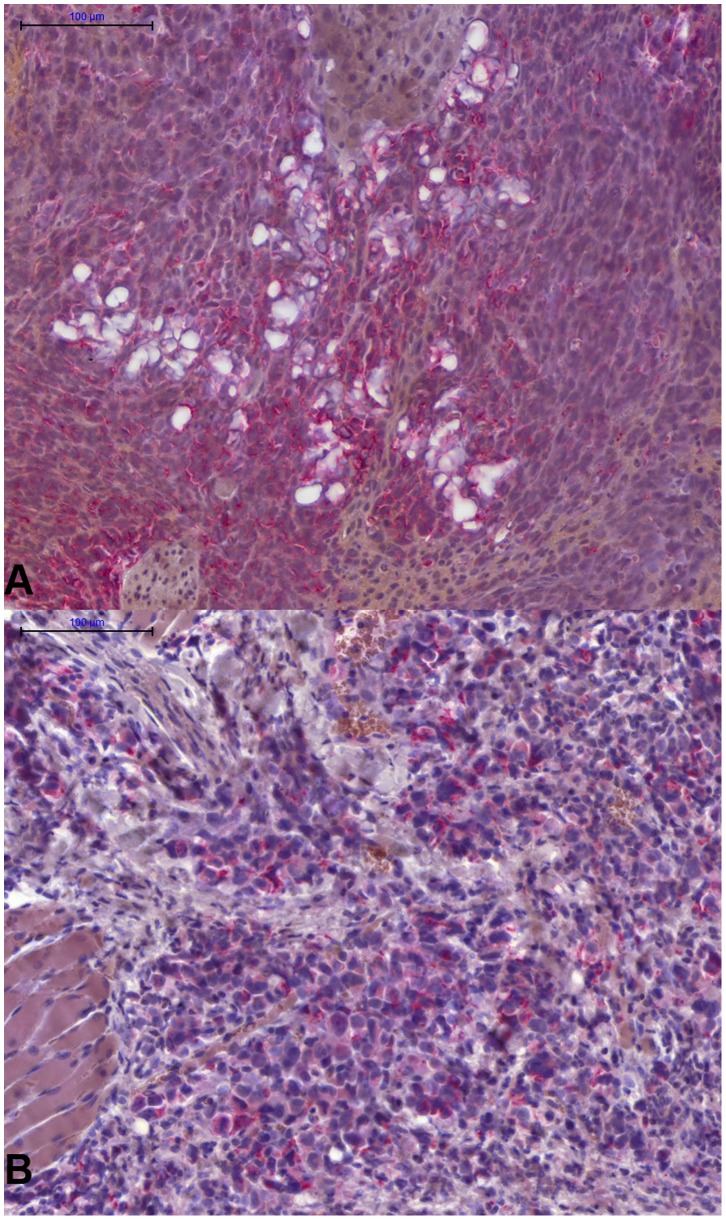
Human CEL and CML cells can be clearly detected by immunohistochemistry in the tissues of the scid mice. **A**: Tissue section of an EOL-1 chloroma of selectin wt scid mouse. Immunohistochemical staining for human HLA-DR in red. **B**: Tissue section of a K562 chloroma of a wt scid mouse. Immunohistochemical staining for human mitochondria in red.

**Figure 4:**
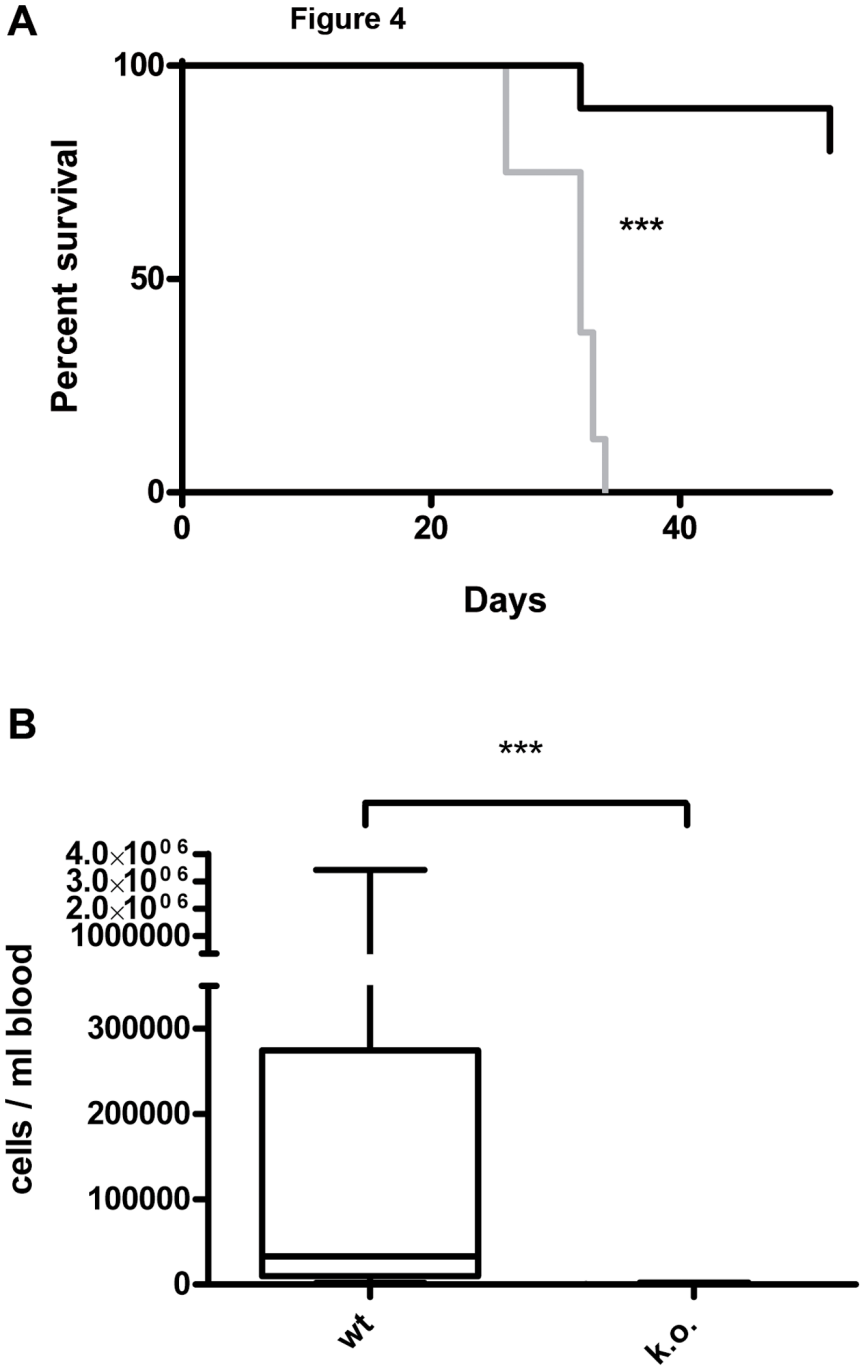
Xenograft model of CEL with the human cell line EOL-1 in wild-type and E- and P-selectin knockout scid mice. Selectin deficiency dramatically increases survival of the animals after injection of 2×10^6^ EOL-1 cells and decreases the number of leukemia cells in the blood. **A**: Kaplan-Meyer survival curve for wild-type (wt, selectin competent, 8 animals, grey curve) and selectin knockout (k.o., E-and P-selectin deficient, 10 animals, black curve). The percentage of surviving animals on a given day is shown. The experiment was terminated after 53 days. Median survival after transplantation was: wt 32 days, k.o. not reached. The curves were significantly different, ***: P<0.0001 (Log-rank test). **B**: Human EOL-1 cells in the animals’ blood at the time of death as determined by quantitative real-time PCR. Selectin competent (wt, 8 animals) are compared with E- and P-selectin deficient mice (k.o., 9 animals). Given in the box plot are the median (line), highest and lowest number of EOL-1 cells per ml of the animals’ blood (whiskers) and upper and lower quartile (box). Median cell number per ml blood was 32950 for the wt group and 7.8 for the k.o. group. The difference between the groups was significantly different, ***: P = 0.0002 (Mann Whitney test).

Two wt mice injected with EOL-1 cells were found dead one day after injection and were excluded from further analyses. All remaining wt animals injected with EOL-1 cells developed varying symptoms of eosinophilic CEL, i.e. cachexia, apathy, palpable tumors/chloroma (**Figure 3A**) and/or paraplegia, and had to be euthanized after 26 to 34 days (median 32 days, **Figure 4A**). This part of the experiment was therefore terminated after 53 days. One mouse of the corresponding k.o. group had to be euthanized after 32 days due to paraplegia (without any further signs of CEL). The animal showed 1605 EOL-1 cells/ml blood, however, necropsy revealed no signs of chloromas and no EOL-1 cells were found in histology. On day 53 only one animal of the k.o. group showed a palpable subcutaneous tumor on the back, but displayed no further signs of CEL and, correspondingly, no median survival can be given for the k.o. group (**Figure 4A**). The resulting survival curves of the wt and k.o. animals for EOL-1 were significantly different (P<0.0001, Log-rank test).

Necropsy and histology revealed multiple organ involvement in the wt group: various animals developed solid chloromas located at the spine (animals showed corresponding paraplegia), in the peritoneum and/or the thorax and showed infiltrations of EOl-1 cells in the bone marrow, liver and/or lung. In the k.o. group, only three animals developed small chloromas in the thorax and only two of these mice showed infiltrations of EOl-1 cells in the liver. No further infiltrations by EOL-1 cells were found in any of the other animals of the k.o. group. Quantitative real time PCR (qRT-PCR) showed a significantly reduced number of EOL-1 cells in the k.o. animals’ blood at the time of death (median of 7.8 EOL-1 cells/ml blood in the k.o. group, range 0 to 2210 cells/ml) compared with the wt group (median of 32950 EOL-1 cells/ml blood, range 1895 to 3.41×10^6^ cells/ml); P<0.0001, Mann Whitney test; (**Figure 4B**).

Summarized, injection of EOL-1 cells caused massive organ infiltration by leukemia cells, chloroma development and massive presence of EOL-1 cells in the animals’ blood in the selectin competent mice. In contrast, small chloromas developed in 30% of the selectin deficient animals, organ infiltration (only liver) was observed in 20% of the selectin deficient mice and only few leukemia cells were detected in their blood. These data suggest an essential effect of the selectins on CEL cell engraftment.

### Selectins are Essential in Leukemia Cell Engraftment in a Xenograft Model of Human CML with K562 Cells

**Figure 5:**
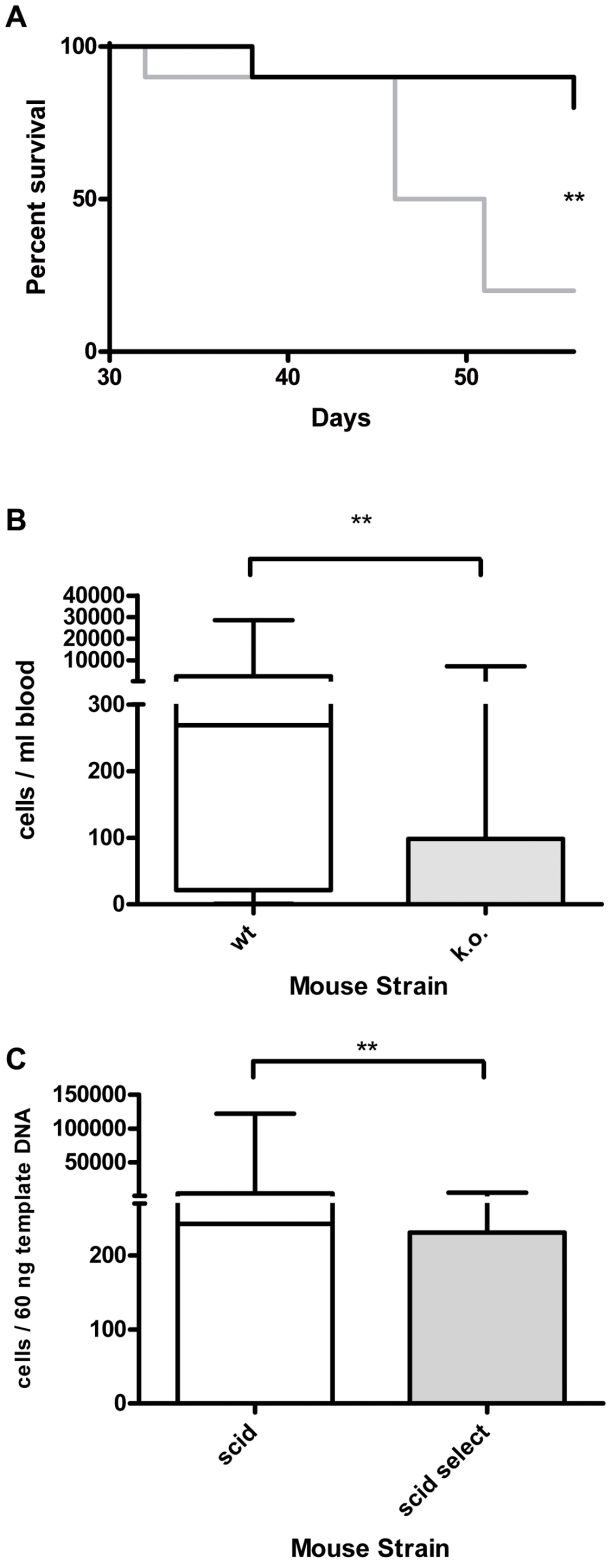
Xenograft model of CML with the human cell line K562 in wild-type and E- and P-selectin knockout scid mice. Selectin deficiency dramatically increases survival of the animals after injection of 2×10^6^ K562 cells and decreases the number of leukemia cells in blood and bone marrow. **A**: Kaplan-Meyer survival curve for wild-type (wt, selectin competent, 10 animals, grey curve) and selectin knockout (k.o., E-and P-selectin deficient, 10 animals, black curve). The experiment was ended after 56 days. Median survival after transplantation was: wt 48.5 days, k.o. not reached. The curves were significantly different, **: P = 0.0085 (Log-rank test). **B**: Human K562 CML cells (given in cells/ml) in the animals’ blood at the time of death as determined by quantitative real-time PCR (qRT-PCR). Selectin competent (wt, n = 10) compared with E- and P-selectin deficient mice (k.o., n = 10). Given in the box plot are the median (line), highest and lowest number of K562 cells per ml of the animals’ blood (whiskers) and upper and lower quartile (box). Median cell number per ml blood was 268.8 for the wt group and 0.2 for the k.o. group. The difference between the groups was significantly different, **: P = 0.0068 (Mann Whitney test). **C**: Human K562 CML cells in the animals’ bone marrow at the time of death as determined by qRT-PCR (given in cells/60 ng template DNA). Given in the box plot are the median (line), highest and lowest number of K562 cells (whiskers) and upper and lower quartile (box). Median cells per 60 ng template DNA in the wt group were 243 compared with 0 cells in the k.o. group. This difference was significant, **: P = 0.0089 (Mann Whitney test).

In mice xenografted with K562 cells, 8 out of 10 animals of the wt group had to be euthanized at some time point during the experiment (it was terminated after 56 days, **Figure 5A**). All of these animals had developed varying symptoms of CML, i.e. cachexia, apathy, palpable tumors/chloromas (**Figure 3B**). In these mice, no paraplegia was observed. Using qRT-PCR, varying amounts of K562 cells were found in these animals’ blood and in the bone marrow of all mice but one. Additionally, six of these animals showed visible facial tumors/chloromas. Two mice had not developed any symptoms until day 56 and both animals did not show any chloromas although a low amount (14.5 cells/ml) of K562 cells was detected in the blood of one of the two mice by qRT-PCR. Additionally, K562 cells were found in this animal’s lung using immunohistochemistry. Thus, only one animal (10%) of the wt group showed no signs of engraftment until the end of the experiment.

In the selectin k.o. group, only two animals developed symptoms of CML until day 56 and had to be euthanized (**Figure 5A**). These two mice were the only ones in this group that displayed facial tumors and other chloromas and were qRT-PCR positive for K562 cells in blood and bone marrow.

Summarized, qRT-PCR results were: Median of 268.8 cells/ml blood (range 0 to 28657 cells) in the wt group compared with a median of 0.2 in the k.o. group (range 0 to 7237 cells) (**Figure 5B**). This difference was significant (P = 0.0068, Mann Whitney test). In the bone marrow of the wt mice, a median of 243 cells/60 ng template DNA was detected (range 0 to 122047 cells) compared with a median of 0 cells/60 ng template DNA (range 0 to 5112 cells) in the selectin k.o. group (**Figure 5C**). Again, this difference was significant (P = 0.0089, Mann Whitney test).

Injection of K562 cells caused massive organ infiltration by leukemic cells, chloroma development and presence of K562 cells in the blood of 90% of the animals in the selectin competent mice. In the selectin deficient animals, only 20% of these mice developed chloromas and showed signs of bone marrow infiltration and only very few leukemic cells were detected in their blood. No further infiltration of other organs was observed in any of the selectin deficient animals. These data suggest an essential effect of the selectins on CML cell engraftment.

### Detection of Selectin Ligands on CEL and CML Cell Lines

In order to identify the E- and P-selectin ligands on cells of the human CEL and CML cell lines, selectin binding after (pre)incubation of cells from culture with potentially inhibiting antibodies was measured by flow cytometry. The selectin binding human pancreatic carcinoma cell line PaCa 5061 was used as a positive control [34].

**Figure 6:**
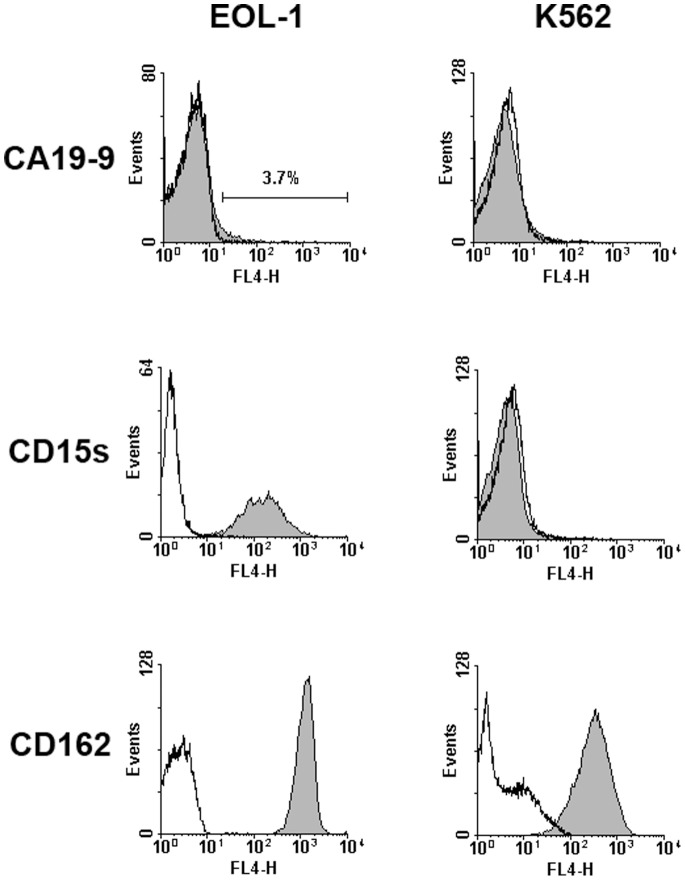
Analysis for potential selectin ligands on the surface of the human CEL and CML cell lines EOL-1 and K562 by flow cytometry. Only EOL-1 cells are positive for sialyl Lewis x, both cell lines are positive for CD162 (PSGL-1). Cells were incubated with antibodies against CA19-9 (sialyl lewis a), CD15s (sialyl lewis x) or CD162 (PSGL-1) or the respective isotype controls followed by an APC-labelled secondary antibody. Given in the histograms are the fluorescence signals and event numbers, labeling with the antibodies against selectin ligands is represented by the filled curves, the respective isotype controls by the open curves. Only a small subpopulation (3.7%) of EOL-1 cells was positive for CA19-9. The cells were either completely negative or more than 95% positive for the other ligands. All experiments were repeated twice, representative results are shown.

Of the described selectin ligands, EOL-1 cells displayed only a small subpopulation of sialyl Lewis a (CA19-9) positive cells (3.7%), but were highly positive for sialyl Lewis × (CD15s), whereas K562 cells were negative for both (**Figure 6**). Both cells lines were positive for CD162 (PSGL-1).

**Figure 7:**
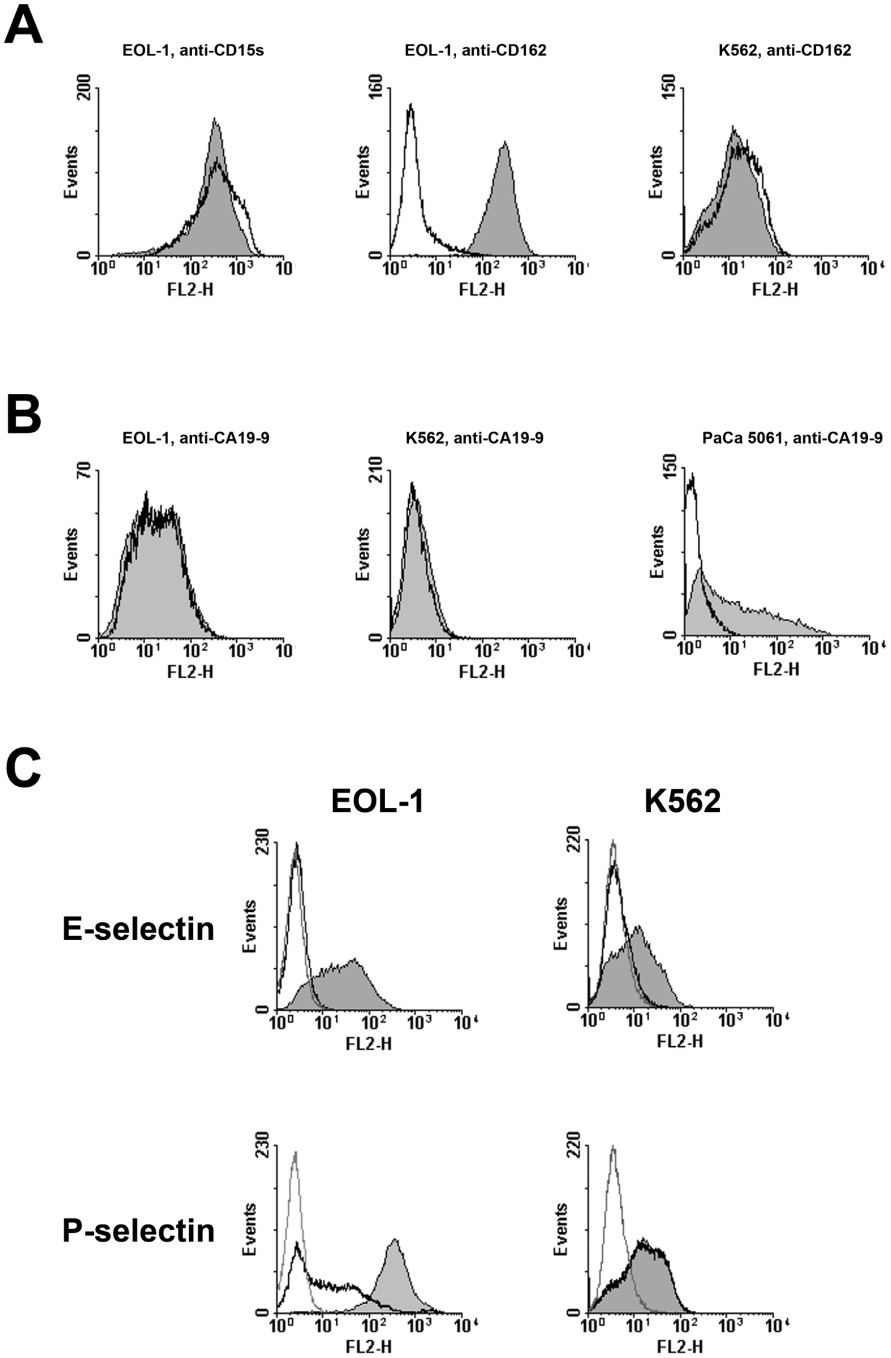
Inhibition of selectin binding to human CEL and CML cells by monoclonal antibodies and neuraminidase treatment as determined by flow cytometry. **A**: Blocking of P-selectin binding by (pre)incubation with monoclonal antibodies against CD15s and CD162. Filled curves in the histograms represent incubation of the leukemia cells with isotype control, open curves represent incubation with the respective specific antibody. The only observed inhibitory effect was caused by anti-CD162 on EOL-1, but not on K562 cells. **B**: Blocking of E-selectin binding by (pre)incubation with monoclonal antibodies against CA19-9. Filled curves in the histograms represent incubation of the cells with isotype control, open curves represent incubation with the specific antibody. The only observed inhibitory effect by anti-CA19-9 was observed on PaCa 5061 pancreatic adenocarcinoma cells (positive control). **C**: Binding of selectins to the leukemia cells after neuraminidase treatment. Filled curves in the histograms: selectin binding without neuraminidase incubation (positive control); open curves, black: selectin binding to neuraminidase treated cells; open curves, grey: negative controls. Neuraminidase treatment abolished E-selectin binding to both cell lines and reduced P-selectin binding to EOL-1 but not to K562 cells. All experiments were repeated twice, representative results are shown.

Antibodies against sialyl Lewis x (CD15s, **Figure 7A**) and sialyl Lewis a (CA19-9, **Figure 7B**) did not inhibit E- and P-selectin binding to EOL-1 and K562 cells although anti-CA19-9 (and not anti-CD15s) did completely block E-selectin binding to PaCa 5061 cells as previously described [34]. Even the monoclonal antibody HECA-452 which has a broader specificity (CA19-9 and CD15s among other carbohydrate moieties) had no inhibitory effect on E- and P-selectin binding to the CEL and CML cells, but blocked E-selectin binding to PaCa 5061 cells (not shown). An antibody against CD162 (PSGL-1) blocked P-selectin binding to EOL-1, but not to K562 (**Figure 7A**), whereas E-selectin binding to both cell lines was not influenced by anti-CD162 (not shown).

As we were unable to confirm that the described selectin ligands sialyl Lewis a and sialyl Lewis x played any role in selectin binding to the CEL or CML line used in our experiments, we treated the cells with neuraminidase (sialidase) to investigate whether sialylated carbohydrate moieties were involved in selectin binding at all. Treatment with neuraminidase abolished E-selectin binding to both EOL-1 and K562 cells and considerably reduced P-selectin binding to EOL-1 cells. However, P-selectin binding to K562 cells was not affected by neuraminidase digestion (**Figure 7C**).

## Discussion

This study was undertaken to analyze the functional role of E- and P-selectin in the process of leukemic dissemination in CML and CEL. Both cell lines used bound to E- and P-selectin fusion proteins: This binding was more intense in EOL-1 cells (moderate to E- and strong to P-selectin) compared to K562 (weak to E- and moderate to P-selectin) and interacted with the selectins under laminar flow conditions. The pathophysiological role of selectin binding could be verified *in vivo*: E- and P-selectin deficient mice showed dramatically increased survival and little organ infiltration and chloroma formation were observed compared with wt mice. Additionally, there were only few to no circulating leukemia cells in the selectin deficient animals’ blood. These observations indicate that E- and P-selectin play an important role in organ infiltration and chloroma formation by CEL and CML cells. Flow cytometric analysis in earlier xenograft experiments with EOL-1 cells showed that nearly all cells vanish from the murine bloodstream after intravenous injection and reappear in the blood about 28 days later [28]. Thus, the human leukemia cells obviously left the bloodstream to home into at least one type of survival niche in the murine organism (in the bone marrow and/or other organs). Whether this niche is similar or identical to the Leukemia Stem Cell (LSC) niche [35], [36] and whether the leukemia cells can establish LSCs in the animals, is a highly pertinent question that can be studied *in vivo* in this model. However, it should be stated that the model applied here employed human cell lines and that cell line-specific LSC may differ from primary LSC in patients regarding their biology and expansion.

Obviously, the leukemia cells were unable to survive in the bloodstream for extended periods of time: In the selectin k.o. animals where the leukemia cells’ ability to leave the bloodstream and enter the organs was impaired, only a minority of the mice showed any sign of tumor cell engraftment at all. This might imply a dynamic situation in human patients as well, with leukemia cells that may constantly be redistributed from and into the bone marrow (and probably also other organs) and adhesion receptors such as E- and P-selectin may play an important role in this redistribution process. Thus, the selectins might be involved in the spread of LSC to new sites in the bone marrow and other organs. Recently, it has been suggested that LSC in acute myelogenous leukemia use the circulation to reach these new sites where they utilize selectins, integrins and other molecules of the leukocyte cascade to leave the bloodstream and subsequently enter their niche again [14]. For hematopoietic stem cells (HSCs) at least two survival niches exist in the bone marrow, as the HSCs are either associated with sinusoidal endothelium or endosteum [37]. Recently, it was demonstrated, that E-selectin is part of the sinusoidal endothelial survival niche itself and that the selectin regulates dormancy and self-renewal of HSCs [26]. If these findings also apply to the survival niche(s) of the EOL-1 and K562 leukemia cells, the selectin deficiency could have effects on both niches: The injected leukemia cells would have a drastically reduced probability of reaching the endosteal survival niche as their ability to leave the bloodstream (adherence to and crossing of the endothelium) is impaired. In the sinusoidal endothelial niche, the lack of E-selectin might prevent the leukemia cells from self-renewal/proliferation. As a result of these two effects, the overall number of leukemia cells in the animals’ bone marrow and blood could have dropped below the detection threshold. Due to the selectin deficiency, the few leukemia cells escaping dormancy in the endothelial niche of the k.o. mice would again encounter great difficulties leaving the bloodstream to invade organs or establish chloromas. Future experiments will have to answer the question if (and then to what extent) a dormancy effect similar to the one described for HSCs [26] is involved in the effects of selectin deficiency in our xenograft model.

With the exception of CD162, we were not able to identify the E- and P-selectin ligands on the surface of the leukemia cell lines used in our experiments by inhibition with monclonal antibodies specific for published selectin ligands. Only an anti-CD162 (PSGL-1) antibody inhibited P-selectin binding to EOL-1, but not to K562 cells. Surprisingly, antibodies specific for sialyl Lewis a (CA19-9) and sialyl Lewis x (CSLEX1) and the antibody HECA-452 (recognizing both carbohydrate moieties) were unable to inhibit E- or P-selectin binding to EOL-1 and K562 cells (which had to be expected in the latter case as K562 cells are negative for sialyl Lewis a and x). In contrast, the antibody against sialyl Lewis a inhibited E-selectin binding to the pancreatic adenocarcinoma cell line PaCa 5061 (used as positive control), as described earlier [34]. It has been known for decades that the presence of sialyl Lewis x alone is not sufficient for a protein to function as a selectin ligand [38] and it has been shown recently that binding of HECA-452 does not block simultaneous E-selectin binding to sialyl Lewis x microspheres [39]. Our results appear to verify the latter finding on a cellular level. It is unlikely, however, that the antibody (CSLEX1) against sialyl Lewis x that we used in this study is similar to HECA-452 in this respect (not able to block simultaneous selectin binding) as it has been shown to block E- and P-selectin binding on a cellular level before [40], [41]. We could show that sialylated carbohydrate moieties are involved in E-selectin binding to EOL-1 and K562 cells, as neuraminidase (sialidase) digestion abolished E-selectin binding to both cell lines. Interestingly, neuraminidase digestion only partially inhibited P-selectin binding to EOL-1, but not to K562 cells where P-selectin binding was unaltered by neuraminidase digestion, indicating that P-selectin binding does not depend on neuraminic acid in the latter case. Other carbohydrate moieties might be critically involved in selectin binding to the leukemia cells, as especially indicated by the results for K562 cells and P-selectin. Taken together, our results suggest that the selectins mainly bind to noncanonical ligands on the CEL and CML cells. Interestingly, it has been shown recently, that binding of HSCs to E-selectin is also mediated by noncanonical selectin ligands [26]. If the EOL-1 and K562 cells display the same (hitherto unidentified new E-selectin ligands) this would give further credit to the assumption that the absence of E-selectin from the endothelial survival niche causes a dormancy effect similar to the HSCs. Taking into consideration that in CEL and CML cell lines the situation with E- and especially P-selectin ligands appears to be more complicated than expected from recent literature on granulocytes – which more or less takes the essential role of sialyl Lewis x for granted [42], [43] – we think that our results certainly warrant further examination of the selectin ligands on other cell lines of CEL, CML and other leukemia entities as well. As leukemia cells display surface expression of markers typical for their normal leukocyte counterparts (e.g. B-lymphoid CLL cells CD19, CD20 etc.) it is more than likely that they express selectin ligands as well [12]. Further experiments will have to include different types of leukocytes to clarify if differences in carbohydrate involvement in E- and P-selectin binding reflect the different progenitor cells of the leukemia cell lines (e.g. eosinophilic granulocyte or monocyte precursors). It will also be essential to know whether primary leukemic stem cells (LSC) derived from patients with CML and CEL express selectin ligands and invade tissues in a selectin-specific manner. Notably, it is well known that LSC in CML interact with several types of niches in various organs, and that LSC-niche interactions contribute to self-renewal and drug resistance [10], [36]. Finally, it is generally appreciated that LSC are important targets of therapy in various types of acute and chronic leukemia [44], [45], [46], [47].
